# Microbial community dynamics alleviate stoichiometric constraints during litter decay

**DOI:** 10.1111/ele.12269

**Published:** 2014-03-17

**Authors:** Christina Kaiser, Oskar Franklin, Ulf Dieckmann, Andreas Richter

**Affiliations:** 1 Evolution and Ecology Program, International Institute for Applied Systems Analysis (IIASA)Schlossplatz 1, A-2361, Laxenburg, Austria; 2 Department of Microbiology and Ecosystem Science, University of ViennaAlthanstrasse 14 A-1090, Vienna, Austria; 3 Ecosystem Services and Management Program, International Institute for Applied Systems Analysis (IIASA)Schlossplatz 1 A-2361, Laxenburg, Austria

**Keywords:** Carbon cycling, ecological stoichiometry, functional microbial groups, individual-based modelling, litter decomposition, microbial community composition, micro-scale spatial structure, nitrogen cycling

## Abstract

Under the current paradigm, organic matter decomposition and nutrient cycling rates are a function of the imbalance between substrate and microbial biomass stoichiometry. Challenging this view, we demonstrate that in an individual-based model, microbial community dynamics alter relative C and N limitation during litter decomposition, leading to a system behaviour not predictable from stoichiometric theory alone. Rather, the dynamics of interacting functional groups lead to an adaptation at the community level, which accelerates nitrogen recycling in litter with high initial C : N ratios and thus alleviates microbial N limitation. This mechanism allows microbial decomposers to overcome large imbalances between resource and biomass stoichiometry without the need to decrease carbon use efficiency (CUE), which is in contrast to predictions of traditional stoichiometric mass balance equations. We conclude that identifying and implementing microbial community-driven mechanisms in biogeochemical models are necessary for accurately predicting terrestrial C fluxes in response to changing environmental conditions.

## Introduction

Theoretical models have suggested that the imbalance between stoichiometry of microorganisms and their substrate is the major cause for C or N limitation of organic matter decomposition (Moorhead *et al*. 1996; Manzoni *et al*. 2008; Manzoni & Porporato 2009). This is based on the idea that microbes have to maintain their elemental composition within certain boundaries, irrespective of the substrate they feed on (Sterner & Elser 2002a; Cleveland & Liptzin 2007). As a consequence they need to either slow down decomposition, if one element is limiting, or increase the efflux of the element that is in excess, for example by N mineralisation or ‘overflow’ CO_2_ respiration (Schimel & Weintraub 2003; Manzoni & Porporato 2009; Manzoni *et al*. 2012). Such a stoichiometric regulation, however, takes place at the level of an individual microbial cell, rather than at the community level. At the community level, resource competition among microbes may lead to community shifts, which may alter the community's carbon and nitrogen use efficiency and thus the overall response of decomposition to resource stoichiometry. While previous studies and models have focused on ‘upscaling’ the physiology of microorganisms to the bulk microbial biomass (Sterner & Elser 2002a; Manzoni *et al*. 2012; Sinsabaugh *et al*. 2013; Wieder *et al*. 2013), the mechanisms that operate at the community level and the community-driven response to stoichiometry have not been explored so far.

At the micro-scale, a microbe's competitive success will likely be determined by the balance between species-specific C : N demand and the local availability of resources (Tilman 1982; Cherif & Loreau 2007). Microbial growth rates and cellular C : N ratios (which both determine the C : N demand) may thus be relevant functional traits with respect to competition. These traits exhibit considerable variations across microbial taxa. The size of a fungal cell, for example, differs by up to three orders of magnitude from that of a bacterial cell (Rutz & Kieft 2004; Bryan *et al*. 2010), and fungi have a profoundly different macromolecular composition compared to bacteria. Cell sizes are often linked to growth rates: Microbes with smaller cell sizes exhibit a higher surface to volume ratio, which allows them to grow faster than microbes with large cell sizes. Slower growing microbes, on the other hand, often have to invest more in structural and/or defensive compounds to compensate for their reduced ability to quickly rebuild biomass (K- vs. r-strategists). Such differences in life-history traits will thus likely influence both competition for C and nutrients and substance flow during litter decomposition. Another important microbial trait is the ability to release extracellular enzymes to breakdown complex substrates into compounds small enough for uptake. Because products of enzymatic deconstruction diffuse in the soil solution and become accessible also to other microbes, not only competitive but also synergistic interactions occur among microbes (Czárán & Hoekstra 2009; Cornforth *et al*. 2012; Johnson *et al*. 2012).

Collective dynamics emerging from such spatial interactions between individual microbes at the micro-scale are likely to reveal unexpected non-linear system properties at the macro-scale (Grimm *et al*. 2005). A new generation of models have started to include micro-scale interactions between individual microbes for investigating microbial decomposer systems (Allison 2005, 2012; Ginovart *et al*. 2005; Folse & Allison 2012), which has shed new light on the role of microbial community dynamics for decomposition. Although some of these models explicitly include stoichiometric C and N fluxes, they did not show (or did not aim to show) a consistent link between C and N availability and community dynamics. The key question thus still remains: how does functional diversity and community dynamics regulate decomposition and its response to litter stoichiometry?

Here, we explicitly address this question with a new model that accounts for micro-scale processes and functional diversity in a way different from previous microbial models. Our model is individual-based, spatially and biogeochemically (C and N) explicit. It is based on microbial functional groups differing in life-history traits, such as cell size and associated maximum turnover rates, biomass C : N ratio and production capacity of extracellular enzymes for degradation of plant or microbial material. The model was calibrated and tested based on published data from a laboratory litter decomposition study (Wanek *et al*. 2010a; Keiblinger *et al*. 2012; Leitner *et al*. 2012; Mooshammer *et al*. 2012). We asked the questions (1) how differences in life-history traits across microbial groups govern community shifts in response to input stoichiometry, and (2) whether changes in community composition have the potential to significantly contribute to the overall response of decay rates to resource stoichiometry.

Our results reveal a strong link between initial litter C : N ratio and functional community dynamics, which in turn influences decay rates in a way that is not predictable from litter stoichiometry and microbial physiology parameters. They further demonstrate that adaptations at the community level in response to resource stoichiometry allow microbes to overcome large stoichiometric imbalances between litter and microbes by controlling the N recycling flow, a result that challenges predictions of established stoichiometric models.

## Material and Methods

### Model description

**Figure 1:**
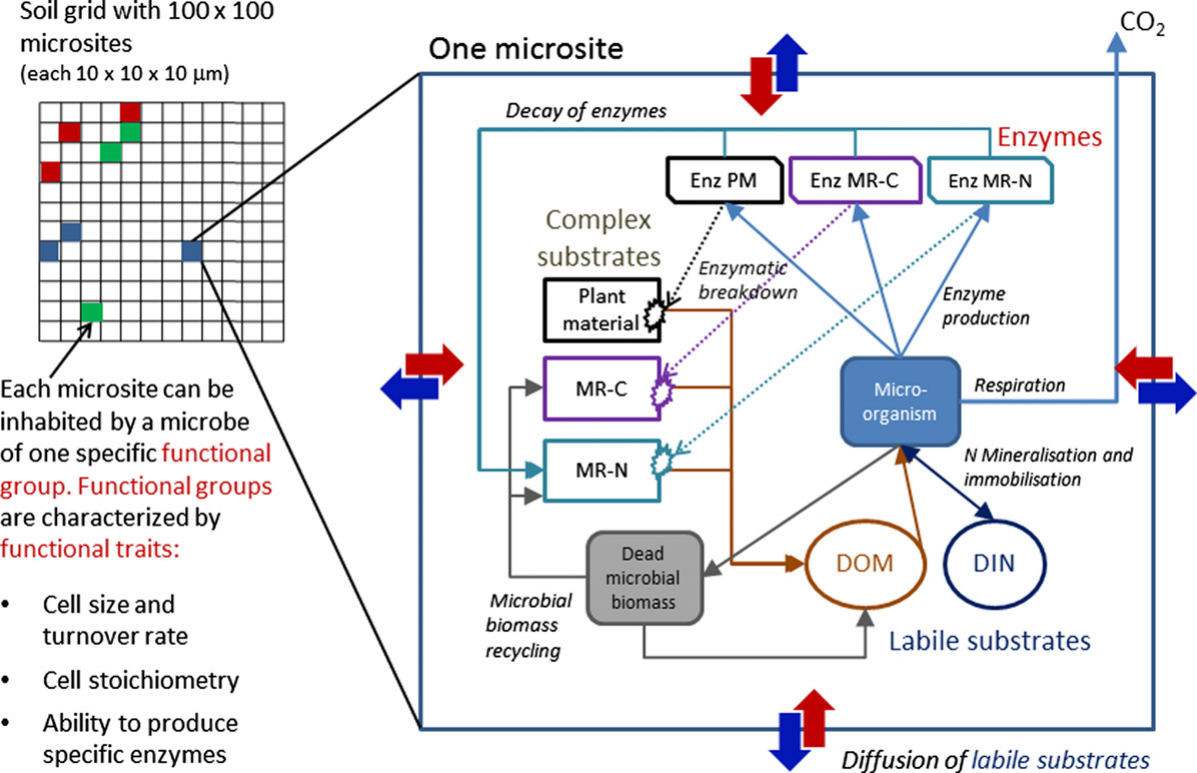
Conceptual diagram of the microbial functional groups model. Solid arrows depict mass flow of C and N. Dotted arrows depict the catalytic effect of a certain enzyme pool on the breakdown of its respective complex substrate (for details on enzymatic breakdown see Appendix S2). Bold blue and red arrows indicate diffusion (for details on diffusion algorithm see Appendix S3). All transformations are calculated once per time step for each microsite on the grid at a random order. MR-C: C-rich Microbial remains (contains cell walls, lipids, carbohydrates, C : N ratio = 150). MR-N: N-rich microbial remains (contains proteins, DNA, RNA from dead microbial cells, and denatured extracellular enzymes, C : N ratio = 5). DOM, dissolved organic material. DIN: dissolved inorganic nitrogen.

We developed a process- and individual-based computer model (programmed in JAVA, using Eclipse IDE, The Eclipse Foundation, Ottawa, Canada, http://eclipse.org/), which aimed at capturing the link among resource availability, microbial community composition and decomposition processes. Each soil microbe is individually represented as one object in the computer memory. Microbes in the model colonise a virtual grid of 100 × 100 soil or litter microsites, each site being 10 × 10 × 10 μm, the whole grid thus representing a piece of 1 mm × 1 mm of soil or decomposing litter. Each microsite can host a single microbial cell or a small colony of cells, depending on the microbe's cell size. Each microsite can only be inhabited by microbes of one functional group. The model runs at discrete time steps (1 time step = 3 h). All biochemical transformations and stoichiometry are calculated explicit each time step for each microsite and executed in a random order across the grid (for a graphical model overview see Figure 1).

### Substrate pools and enzymatic degradation

Each microsite can hold up to three pools of complex substrate, one ‘primary’ (= original plant material) and two ‘secondary’ substrates (= complex compounds that accumulate as a result of microbial activity). Secondary substrates are either C-rich microbial remains (contains cell walls, lipids, carbohydrates and others, with a C : N ratio of 150) or N-rich microbial products (contains proteins, including denatured extracellular enzymes, DNA and RNA, C : N ratio = 5). Each of the three complex substrate pools is associated with one class of enzymes, which catalyses its degradation. Enzymatic breakdown is calculated each time step according to Michaelis–Menten kinetics (Appendix S2) (Allison *et al*. 2010; Wang & Post 2013). Products from enzymatic degradation of complex substrates are added to the dissolved organic matter (DOM) pool at the respective microsite. In addition, each microsite has a pool of dissolved inorganic nitrogen (DIN). DOM and DIN move via diffusion between neighbouring microsites in each time step (Appendix S3). A certain fraction of DOM and DIN that is diffusing between microsites is lost by leaching.

### Microbial processing of C and N and stoichiometric overflow

Microbes take up C and N in the ratio present in the DOM pool at their microsite at a rate related to cell surface area (Appendix S1). Maintenance respiration is calculated as a fraction of microbial biomass per time. A small part of enzymes (one tenth of maximal enzyme production) are produced constitutively in our model, i.e. also under conditions where uptake is not sufficient to cover maintenance respiration. If maintenance respiration and constitutive enzyme production exceed C uptake, biomass decreases (starving). After deduction of maintenance respiration and constitutive enzyme production, absorbed C and N are used for enzyme production and growth. If needed, microbes additionally take up N from the DIN in their microsite (N immobilisation). Growth and enzyme production require additional respiration, which is calculated as a fraction of the C used for the respective process (Schimel & Weintraub 2003). The model assumes that microbes need to keep their cellular C : N ratio constant. After meeting the needs for enzyme production and growth, the element in excess is thus released, either by N mineralisation (transfer of excess N into the DIN pool) or by overflow C respiration (Schimel & Weintraub 2003; Manzoni *et al*. 2012).

### Mortality and reproduction

Microbes can die by starving (if biomass falls below a minimum limit), or due to ‘catastrophic death’ (reflecting predation or abrupt changes in environmental conditions). The latter is implemented as a functional-group specific probability of each individual to die in each time step. This stochastic mortality randomly creates empty microsites, which will be occupied by the most successful microbes in their surrounding: Microbial cells divide and colonise a neighbouring microsite if their biomass exceeds a functional-group specific maximum level. If all neighbouring microsites are occupied, microbes can ‘invade’ an occupied microsite with a probability of 0.01 (leading to the death of the owner). Microbes are not ‘mobile’ in the model. The only movement of microbes on the grid is due to dispersal to neighbouring microsites in the course of reproduction. Upon cell death, microbial cellular compounds are distributed among the substrate pools of the microsite (C-rich and N-rich microbial remains, and DOM). After a defined lifetime, extracellular enzymes will also be shifted to the N-rich microbial remains pool which makes them inactive and ready to be degraded by other enzymes.

### Functional traits

A functional microbial group is composed of microbes with certain functional traits. Functional traits can be classified in ‘life-history’ traits (microbial cell sizes and associated turnover rates, cell C : N stoichiometry and the microbe's investment in enzyme production) and ‘substrate-specificity’ traits (the ability to produce specific enzymes to degrade specific substrates).

1. Cell size and turnover rates: Each functional group is characterised by its ‘maximum cell size’; (MCS), which is the cell size at which a microbe divides and colonises neighbouring cells. All individuals of a functional group will thus have cell sizes distributed between half of MCS and MCS. We coupled cell size and growth rates in the model by relating maximum possible uptake rates to the cell surface : volume ratio. In addition, we assumed that species with larger cells are more resistant against catastrophic death, thereby we related (stochastic) mortality rate inversely to MCS. Actual growth rates in our model are thus a result of (i) local availability of labile C and N and (ii) functional-group specific maximum uptake and mortality rates. (Appendix S1).
2. Cell chemical composition and C : N ratio: Each functional group is characterised by its cell stoichiometry and chemical composition (Table 1). Microbial biomass is divided into three compartments: (i) low molecular weight substances (sugars, amino acids or small peptides, C : N ratio 15) (ii) N-rich macromolecules (proteins, DNA, RNA, C : N ratio 5) (iii) C-rich structural or storage compounds (cell wall compounds, carbohydrates, lipids, C : N ratio 150). The ratio of this three pools determines the overall C : N ratio of the cell (Table 1).
3. Production of extracellular enzymes: Each functional group is characterised by the fraction of C uptake invested into enzyme production and by which types of enzymes it can produce.

**Table 1:** Microbial cell stoichiometries, cell sizes and enzyme production rates used for the evaluation of the effect of life-history traits on resource competition (Fig. 4)

Parameter	Description	r-strategist (bacteria)	Generalist (bact/fungi)	K-strategist (fungi)	Unit
Cell stoichiometry*	Cell component	Fraction of cell biomass			
Cell DOM	Cell solubles, immediately available for uptake by other microbes upon cell death (C : N ratio = 15)	0.06	0.06	0.06	
Cell MR-C	C-rich microbial remains, i.e. cell wall compounds, lipids, starch (C : N ratio = 150)	0.78	0.52	0.37	
Cell MR-N	N-rich microbial remains, i.e. proteins, DNA, RNA (C : N ratio = 5)	0.16	0.42	0.57	
Resulting cell C : N ratio		**6.21**	**9.03**	**12.22**	
**Cell size and turnover rates**†		**Fast**		**Slow**	
Max cell size	Size at which a microbial cell divides and colonises a neighbouring microsite	10		100	fmol C
Max cells ms	Max. number of microbes of that group in one microsite	3		1	
**Enzyme production**		**Half**		**Full**	
Enz fract	Fraction of microbial C uptake after deduction of maintenance respiration that is invested in enzyme production	0.06		0.12	
Enz ratio	Ratio in which specific enzymes are produced for the degradation of plant material: C-rich microbial remains: N-rich microbial remains	0.7: 0.15: 0.15		0.7: 0.15: 0.15	

Cell stoichiometries presented here have also been used as assumptions for the three distinct functional groups used in the Bayesian calibration of the model.

* Chemical composition of prokaryotic and eukaryotic (e.g. yeast) cells based on Kirchman (2012).

† Microbial cell sizes based on Rutz & Kieft (2004); Clode *et al*. (2009); Bryan *et al*. (2010); Romanova & Sazhin (2010). Turnover rates are cell size dependent because: (1) growth is related to cell size based on the assumption that uptake is surface dependent and smaller cells have a larger surface to volume ratio, i.e. smaller cells grow relatively faster compared to larger cells. (2) Mortality rate is inversely linked to maximum biomass per microsite, assuming that larger cells invest more in defensive structures. For more details, see Appendix S1.

### Model calibration with experimental data

**Figure 2:**
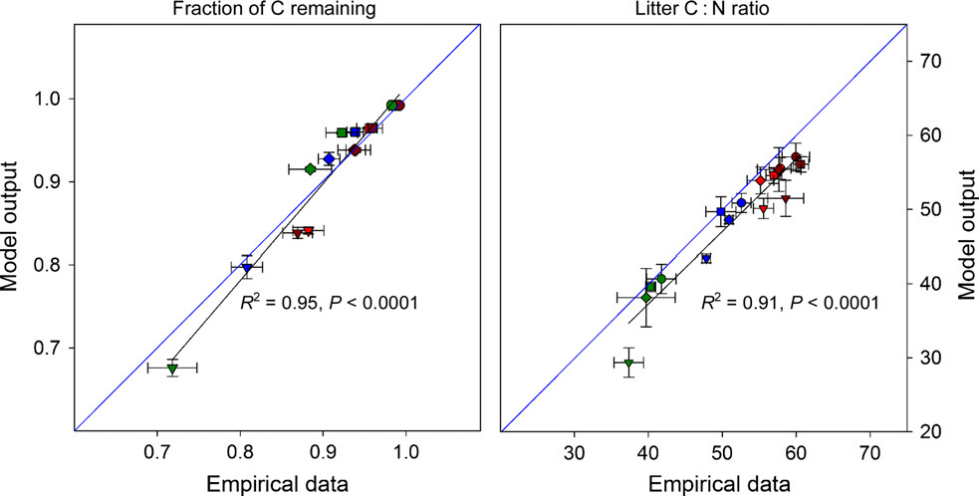
Comparison of model output with data from a litter incubation study (Wanek *et al*. 2010; Mooshammer *et al*. 2012). Beech litter of four sampling sites in Austria differing in litter stoichiometry had been incubated for up to 65 weeks. Mean (± standard error) of initial litter C : N mass ratios from distinct sampling sites were as follows: 41.8 ± 0.8 (indicated by green symbols), 52.6 ± 0.5 (blue), 57.9 ± 0.6 (red) and 60.0 ± 0.7 (dark red). Incubation times/model time of 2, 14, 26 and 65 weeks is represented by circles, squares, diamonds and triangles respectively. Remaining carbon is calculated from measured respiration rates during the litter decomposition study. Model parameter settings are given in Table S1. The model was run in five replicates for each of the initial litter C : N ratios of the study. Empirical data from incubations at initial litter 41.8 and 60.0 (green and dark-red symbols) had been included in the model calibration (Markov Chain Monte Carlo).

We calibrated the model parameter with a Bayesian approach [Markov Chain Monte Carlo simulation (Van Oijen *et al*. 2005)] using empirical data from a beech litter decomposition experiment (Wanek *et al*. 2010b; Mooshammer *et al*. 2012). (Appendix S4). After calibration, the model predicted overall C and N dynamics of the empirical study reasonably well (Fig. 2). Other C and N pools and fluxes that are calculated by the model (such as gross N mineralisation, microbial respiration, DIN) were in the same order of magnitude as in the empirical study.

### Evaluating the effect of life-history traits on competition

To evaluate how differences in life-history traits between microbial groups drive community shifts in response to input stoichiometry, we run the model with two functional groups at initial litter C : N ratios from 15 to 95. One of the two groups had fixed traits (C : N ratio = 12, MCS = 100 fmol C, enzyme production = 12% of C uptake after deduction of maintenance respiration), whereas the second group's functional traits were systematically varied (C : N ratio = 6, 9 or 12; MCS = 100 fmol C or 10 fmol C, roughly corresponding to fungal and bacterial cell sizes; enzyme production = 12% or 6%). As MCS is linked to uptake and mortality rates, MCS = 100 and 10 fmol C correspond to slow and fast turnover rates respectively. Microbes of all groups produced all three extracellular enzymes present in the model at the same ratio (7 : 1.5 : 1.5 for enzymes degrading plant material: C-rich remains: N-rich remains).

### Evaluating the effect of substrate-specific enzyme production

For evaluating the link between community and substrate dynamics during litter decay, we ran the model with three functional groups. ‘Plant degraders’ and ‘microbial-remains degraders’ both produce extracellular enzymes at a rate of 12% of the C uptake remaining after maintenance respiration. Ninety per cent of enzymes produced by ‘plant degraders’ specifically degrade plant material, whereas 10% degrade N-rich microbial remains. ‘Microbial-remains degraders’ produce enzymes degrading C-rich and N-rich microbial remains (each 50% of total enzyme production). ‘Opportunists’ were fast-growing microbes without any enzyme production capabilities, assuming this a realistic component of the microbial decomposer community. We ran the model at four different initial litter C : N ratios (from 45 to 75) and recorded the C : N ratio of total dissolved organic material (aggregated over the grid) as a measure of microbial C : N limitation at any time point. We calculated community carbon use efficiency (CCUE, i.e. the fraction of the total carbon uptake that is used for microbial growth) as an emergent result from the output of the model in each time step as:


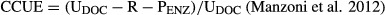


where U_DOC_ is the total amount of DOC taken up by all microbes on the grid, R is the total amount of C respired and P_ENZ_ is the total amount of C released as extracellular enzymes.

## Results

### Emerging spatiotemporal dynamics of microbes and resources

**Figure 3:**
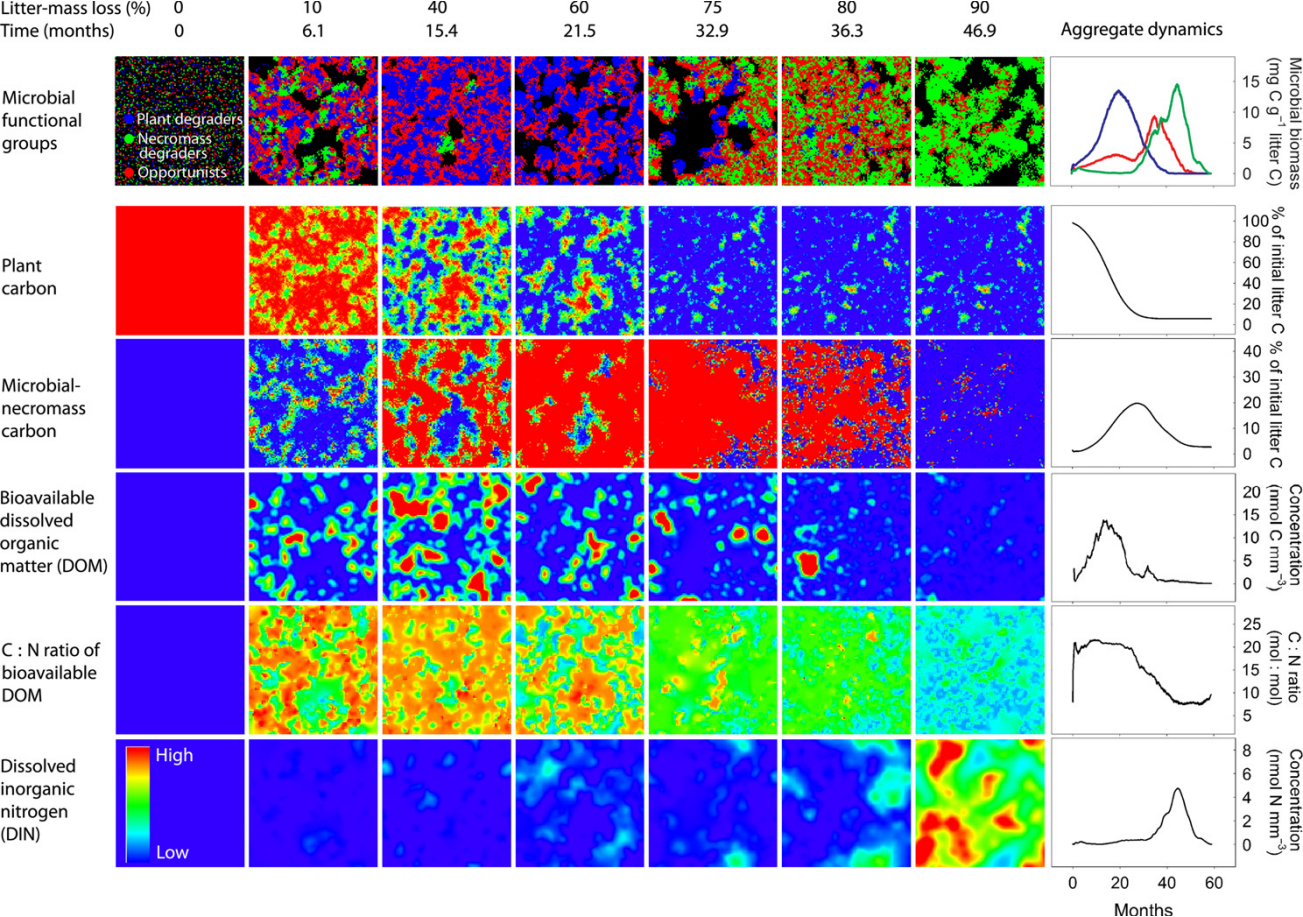
A closely linked succession of microbial groups and chemical complexity emerges during litter decomposition from individual-based modelling. Each square shows a 100 × 100 grid of microsites, corresponding to a 1 × 1 mm area of leaf litter. The uppermost row displays the spatial distribution of individual microbes of three functional groups (blue: Plant degrader, green: Microbial-necromass degrader, red: Opportunists), whereas rows 2–6 display spatial distribution of complex (plant material, microbial remains) and labile substrate (dissolved organic material, inorganic nitrogen). Relative amounts of each substrate per microsite are indicated by the colour code in the lower left corner. Inserted figures on the right show the aggregated sizes of the respective pools as calculated from the model output. For detailed parameter settings, see Table S1 and main text.

Individual-based modelling allows overall system behaviour to emerge as a consequence of the sum of individual behaviour and interactions (Grimm *et al*. 2005; Hellweger & Bucci 2009). In our model, micro-scale competition between microbes with different functional traits promotes a continuous feedback between local resource availability and community composition, which leads to the emergence of closely coupled spatiotemporal dynamics of microbes and litter chemistry (Fig. 3). Spatial heterogeneity of initially uniform distributed substrates increases during a litter decomposition model run: primary substrate (dead plant material) becomes depleted at parts of the grid with high microbial activity, while at the same time microbial products, such as dead microbial cells or remains of enzymes, are accumulating elsewhere (Fig. 3, Movie S1). Diffusion of labile C and N, which are produced at certain spots (by enzyme activity or cell death) and removed at other spots (by microbial uptake), establishes spatial gradients of C and N availability (Fig. 3, Movie S1).

### Effect of life-history traits on microbial community dynamics

**Figure 4:**
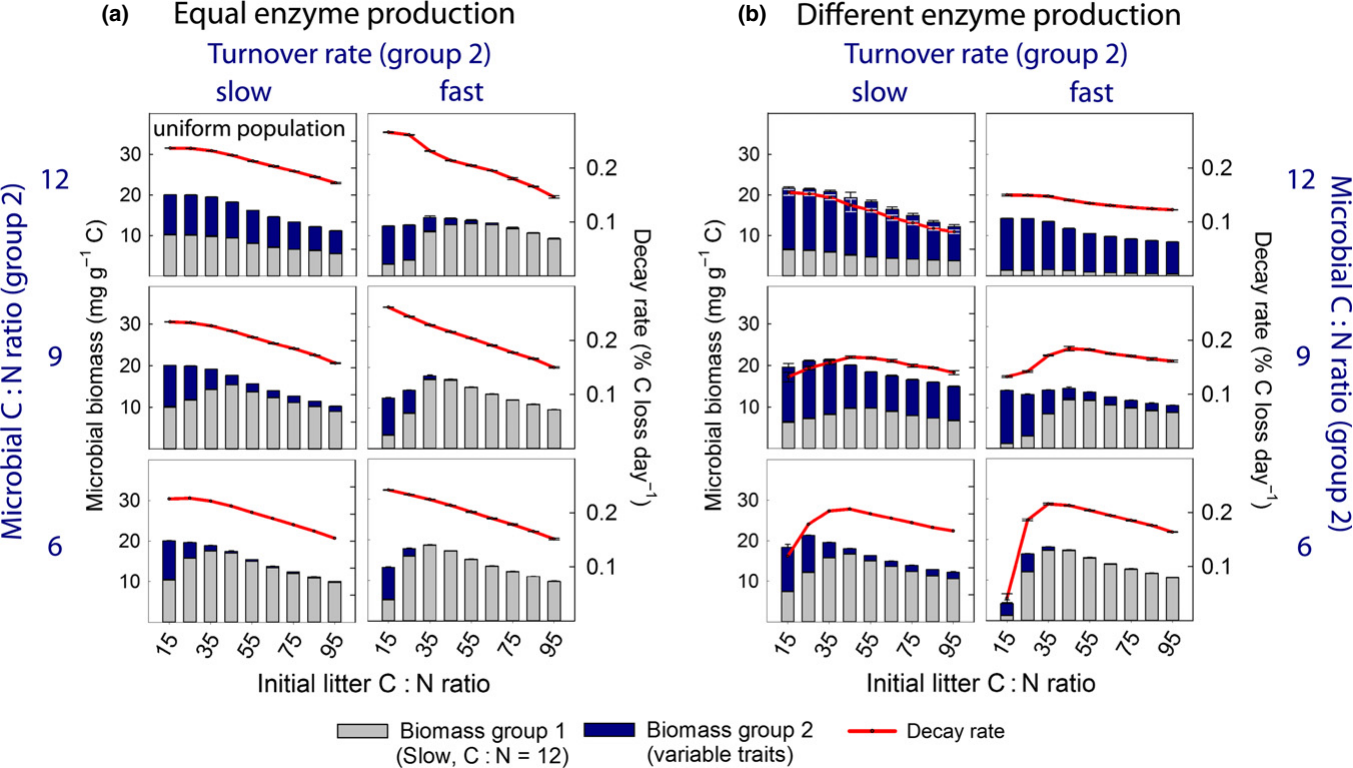
Resource stoichiometry controls competition between microbial groups with different life-history traits, which in turn govern litter decay rates. Displayed are average microbial biomass and decay rates over model runs from 0 to 60% mass loss. Bars (microbial biomass) and dots (decay rates) are means of five replicate model runs (error bar = standard error, sometimes too small to see). Colours within each bar depict proportions of the competing groups (grey = group 1, blue = group 2). Traits of group 1 were kept constant (C : N ratio = 12, turnover rate: slow), whereas group 2 varied as outlined in the figure. The upper left panel thus shows model dynamics with a uniform population (both groups have equal traits). (a) Groups invest the same fraction of uptake into enzyme production. (b) Group 2 invests only half as much as group 1 into enzyme production, all other parameters are identical. For parameter settings see Table 1.

Litter stoichiometry controls community dynamics of two functional groups, when they differ in basic life-history traits. Given that competing microbes invest the same fraction of the substrate taken up into extracellular enzyme production, microbial groups with smaller cell sizes (faster biomass turnover) and/or a lower cellular C : N ratio are only competitive in degrading low C : N litter (Fig. 4a). Slow-growing microbes, on the other hand, are not as efficient at low litter C : N, but can cope relatively better with N limitation. Overall, decay rates are negatively correlated with litter C : N, when competing microbes invest the same fraction into extracellular enzyme production (Fig. 4a).

The situation changes, however, when functional groups invest different fractions of their uptake into enzyme production (‘different enzyme production’, Fig. 4b). When microbes of one group invest only half as much in extracellular enzyme production compared to the other group, they automatically benefit from their competitors activities. This ‘cheating’ increases competitive advantage at all litter C : N ratios (Fig. 4b).

Thus, in scenarios with unequal enzyme production, the effect of litter C : N ratios on community dynamics is no longer predictable from ‘conventional’ microbial stoichiometric traits. In addition, decay rates need no longer be positively correlated with litter N content: when the group that produces less enzymes, for example, has a higher competitive advantage at low litter C : N ratio (e.g. by exhibiting a faster turnover rate, or a lower cellular C : N ratio), it substantially slows down the growth rate of enzyme producers, leading to a reduction in litter decomposition particularly at low litter C : N ratios (Fig. 4b).

### Effect of community dynamics on carbon and nitrogen fluxes

**Figure 5:**
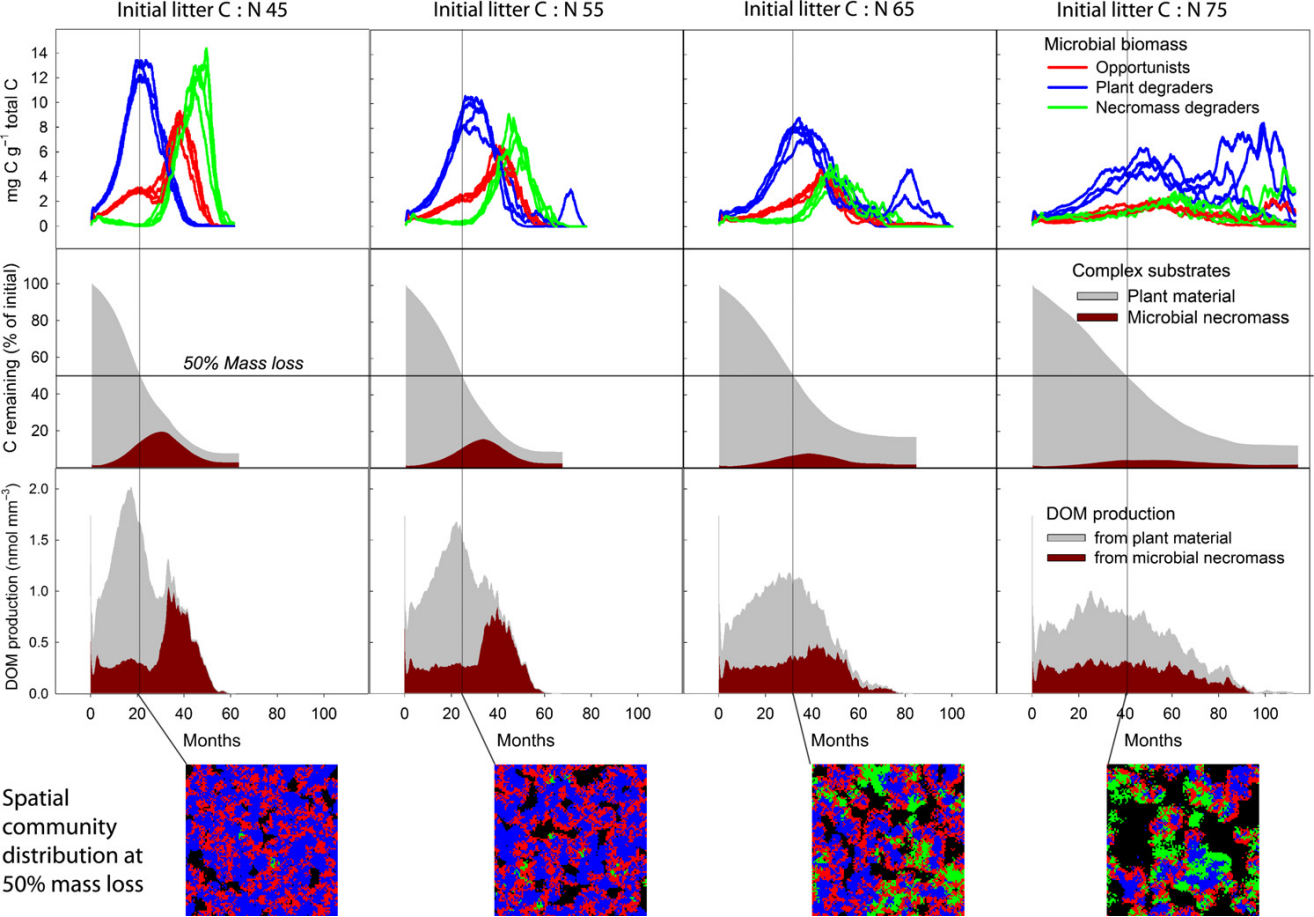
Initial litter C : N ratio affects community dynamics of substrate-specific functional groups, which in turn influences dynamics of complex and labile compounds during litter decay. The model was run at four different initial litter C : N ratios (containing the same amount of carbon, but different amounts of nitrogen in the native plant material) until 90% mass loss. First row: coloured lines show biomass of functional groups over time (for visualising stochastic variability, each panel shows results of four model runs). Group traits as in Figure 1. Second row: Grey and brown areas show remaining C in plant compounds and microbial necromass respectively. Third row: Grey and brown areas show enzymatic DOM production from plant material and microbial necromass respectively. Vertical lines depict the time point of 50% mass loss for each of the four scenarios. Screenshots show the spatial distribution of microbes on the 100 × 100 microsites grid at 50% mass loss.

For a second analysis we implemented functional groups, which did not only differ in basic life-history traits but also in their abilities to deconstruct different substrate polymers. The model was run with three functional groups: one specialised for degrading primary substrates (plant-derived material), one for secondary substrates (microbial remains) and the third was an opportunistic, fast-growing group, without the ability of producing extracellular enzymes. The presence of substrate-specific groups leads to the emergence of a microbial succession during litter decay, with plant degraders peaking before microbial remain degraders (Fig. 3 and 5).

The initial litter C : N ratio substantially affects these dynamics. In low C : N litter, a two-phased decomposition pattern emerges. Initially, plant degraders grow to high numbers, favoured by relatively high plant N concentrations. Degraders of microbial remains only grew to numbers after a significant amount of plant material had been depleted, utilising the meanwhile accumulated microbial necromass (Fig. 3 and 5).

Litter with high initial C : N ratios, by contrast, exhibited a less pronounced succession between the two groups. In this case, microbial-remains degraders exhibited a higher relative abundance already in the early phase of decay. The ratio of degraders of plants: microbial-remains degraders were 35.5 (SE = 8.7, *n* = 4) and 4.2 (SE = 0.3, *n* = 4) for litter C : N = 55 and C : N = 75 at 50% C loss respectively (Fig. 5). The higher relative abundance of degraders of microbial remains at litter with higher C : N ratios is caused by lower competitiveness of the plant-degrading group (due to the poorer N quality of their substrate). This allows degraders of microbial remains to be more competitive at early stages of decomposition, and to degrade emerging patches of microbial remains soon after they appear.

Such changes in community dynamics induced by initial substrate stoichiometry, in turn, influence the dynamics of complex and labile compounds. The two-phase successional community dynamics at low litter C : N ratios initially lead to a strong accumulation of microbial remains, which are degraded in the second stage. DOM in the second stage is thus characterised by an increased contribution from N-rich microbial compounds (C : N ratio ∼10), leading to a significant drop of C : N ratio of DOM at later stages of decay (Fig. 5).

At high litter C : N ratio, by contrast, the higher relative abundance of microbial-remains degraders in the early phase of decomposition lowers the accumulation of microbial necromass over time. At the same time, however, it increases the relative proportion of N-rich microbial necromass to the continuous DOM production (Fig. 5). This result is caused by an interesting emerging mechanism which drives the model dynamics: N is locked up to a greater extent in complex microbial remains in the low C : N litter (i.e. when N is not limiting), but is rather kept in circulation when the C : N ratio of the litter is high, due to a greater activity of microbial recyclers. This leads to a more efficient use, i.e. a faster recycling of N in the high C : N litter.

**Figure 6:**
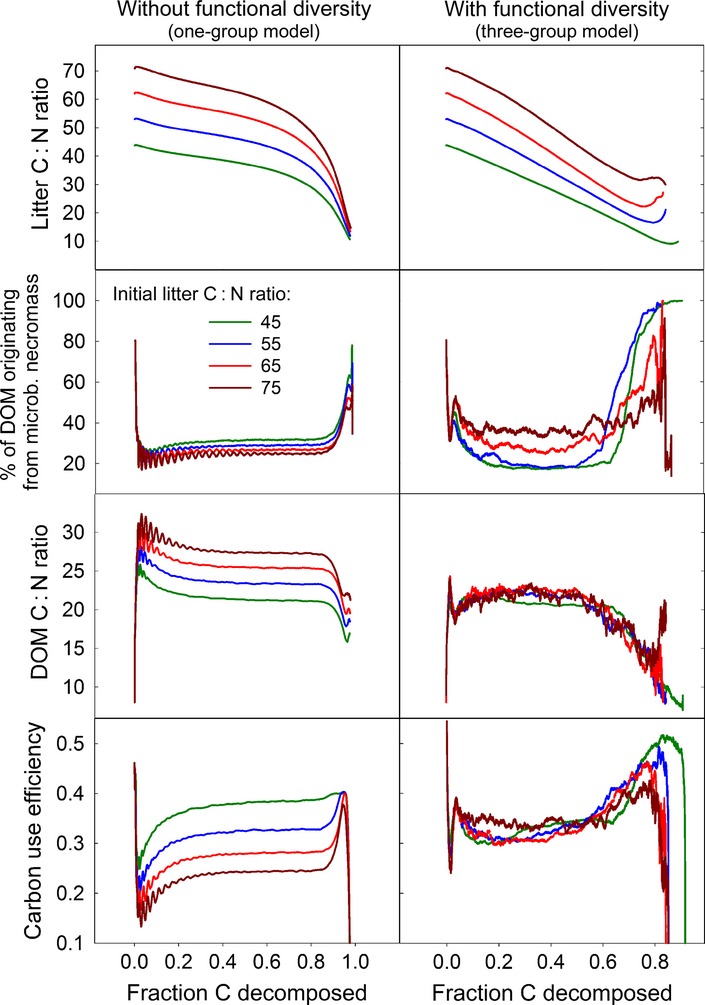
The implementation of community dynamics in the model enables a flexible response of decomposition to stoichiometric conditions. Comparison of outputs of model runs at four different initial litter C : N ratios of (1) a model with a uniform population (left column, all microbes have the same traits and produce all kind of enzymes) and (2) a model including three substrate-specific functional groups (right column, same parameters as in Figure 3). In the one-group model, DOM C : N ratio happens to be coupled to litter C : N ratio, leading to a greater N limitation of microbes in the low-N litter. In the functional diverse model, the system responds to increasing litter C : N ratios with increasing the fraction of DOM from recycled microbial necromass. This lowers DOM C : N ratios, which in turn alleviates microbial N limitation and thereby increases community CUE. For parameter details, see Table S1.

The positive feedback loop between litter C : N and the abundance of degraders of microbial remains leads to the surprising observation that the community composition effectively buffers the C : N ratio of DOM. Independent of the initial litter C : N ratios the C : N ratio of DOM converges to around 22 in all scenarios when the substrate-specific functional group model was used. By contrast, when the model was run without functional group diversity (i.e. all microbes belonged to the same functional group which equally produces all kinds of enzymes), C : N ratios of DOM were still positively correlated with litter C : N ratios (Fig. 6), varying between 22 (for litter with C : N 45) and 28 (for litter with C : N 75). In both models, DOM C : N ratios were at least 50% lower than litter C : N ratios. The surprising decoupling of DOM and litter C : N ratios in our community model is also supported by empirical data from a litter decomposition study where beech leaf litter from four different sampling sites in Austria (varying in initial C : N ratios) had been incubated under laboratory conditions for up to 65 weeks (Wanek *et al*. 2010; Leitner *et al*. 2012; Mooshammer *et al*. 2012). Results from that study show that (1) C : N ratio of dissolved material is constantly lower than C : N ratio of litter and (2) C : N ratio of dissolved material is decoupled from C : N ratio of litter (it only decreases with time, but is independent of litter C : N ratio) (Fig. S1).

By altering DOM C : N ratio, the community-driven mechanism in our model also affected CCUE. This is because individual microbial CUEs are linked to the C : N ratio of available DOM via the implemented mechanism of overflow respiration (N limitation causes overflow C respiration, which decreases microbial CUE). As DOM C : N ratios levelled off at a relatively low value, CCUE converged consequently at a relatively high value of 0.32, independent of initial litter C : N ratios in the community-driven model (Fig. 6).

## Discussion

Microbial community dynamics has so far been neglected in modelling decomposition processes in terrestrial systems due to the difficulty of establishing a link between microbial community structure and function. Our model links C and N fluxes to microbial community dynamics in a bottom-up modelling approach, based on functional microbial groups that differ in life-history traits and substrate-specific enzyme production capacity. Our work shows that (1) simple differences in microbial life-history traits, such as cell size, turnover rates and chemical composition, are sufficient to cause community shifts in response to resource stoichiometry and (2) that community dynamics of specific functional groups regulate the overall response of litter decay to stoichiometry in a way not predictable from the simple imbalance of litter and microbial biomass stoichiometry.

Although microbial decomposer communities are highly diverse, they are also highly redundant with respect to ecological functions, especially at the species level (Prosser 2012). Fundamental functional differences between microbes may, however, occur at higher phylogenetic levels (Philippot *et al*. 2010; Schimel & Schaeffer 2012), the most significant ones probably between fungi and bacteria. Here, we accounted for these differences by considering the large variation in cell size, chemical composition and enzyme production capacities found across microbial phyla. Our results show that resource stoichiometry has the potential to influence microbial community composition based on the presence of contrasting life strategies, such as those broadly assigned to major fungal and bacterial lineages.

Cellular C : N ratios of microbes have often been regarded crucial for microbial competition for C and N resources (Sterner & Elser 2002b; Cherif & Loreau 2007). Our model results suggest that not only C : N ratios but also species-specific turnover rates of microbes may affect competitive success at certain resource C : N ratios. Microbes with faster turnover rates grow faster *and* have higher mortality rates. They therefore have a higher N demand per unit of time, compared to slower growing microbes, which can conserve N in their biomass for a longer time. It is interesting to note that this basic link between turnover rates and the C : N demand is effective even independent of cellular C : N ratios (Fig. 4). In reality, however, cellular C : N ratios and turnover rates are often coupled because slower growing K-strategists invest more in C-rich structural compounds, whereas fast-growing microbes have a larger amount of N-rich ribosomes.

Both high cellular C : N ratios and relatively slow turnover rates yield a competitive advantage at high-substrate C : N ratios in our model. Our results from scenarios with equal extracellular enzyme production (Fig. 4a) thus seem to support the frequently applied ‘rule of thumb’ that (often fungal) K-strategists perform better at high-substrate C : N ratio, whereas fast-growing (often bacterial) r-strategists dominate at low-substrate C : N ratio (Fig. 4a). In reality, however, and despite that this is not considered in most models, it is very unlikely that different microbial groups have an equal share on total extracellular enzyme production. Our results from scenarios with unequal enzyme production show that this mundane fact may substantially change the expected outcome of competition. Fungi, which are thought to be the main producers of extracellular enzymes for plant litter breakdown (Osono 2007; Gessner *et al*. 2010; Voříšková & Baldrian 2013) may thus not necessarily be more competitive at high litter C : N ratios than bacteria, which invest far less in enzyme production (Romaní *et al*. 2006; Schneider *et al*. 2012). Although our results indicate interactions between resource stoichiometry and the dynamics of microbial K- and r-strategists, we did not account for specific fungal-related traits, such as the ability to translocate nutrients via hyphae, nor for a possible antibiotics production of enzyme producers. Such features may additionally affect resource-driven competition between fungi and bacteria.

Although the importance of microbial remains as a precursor for soil organic matter formation is widely acknowledged (Schmidt *et al*. 2011; Wickings *et al*. 2012), the build-up and recycling of this pool during litter decomposition has only been considered in a limited number of litter decomposition models (e.g., Moorhead & Sinsabaugh 2000; Ingwersen *et al*. 2008). Our results indicate that a substantial proportion of the overall C and N flux at any given time during litter decomposition may originate from reprocessing of microbial necromass, explaining why the C : N ratio of DOM can be constantly lower than the bulk C : N material (Fig. 6 and S1). This has far-reaching implications for the concept of stoichiometric limitation of microbes. Stoichiometric limitation of microbial growth is controlled by the C : N ratio of the bioavailable DOM, rather than by the C : N ratio of the overall (complex) substrate. Bioavailable DOM, in our model as in reality, unites inputs from different complex sources, its C : N ratio is thus a result of the ratio of supply rates from different complex substrates with different C : N ratios. If substrates with different C : N ratios, such as a plant-derived and a microbial-biomass derived pool, turnover at different speeds, the rates of C and N recycling in the remaining litter decouple. Recycling C and N at different rates has an instantaneous effect on the ratio at which they are available for microbes at any time point. The C : N ratio of dynamically available compounds in the DOM may thus be fundamentally different from the overall C : N ratio of the substrate.

This concept is not included in state-of-the-art stoichiometric models, which are based on the stoichiometric mass balance between the (complex) substrate and the microbial biomass. For example, the critical litter C : N ratio (or threshold element ratio, TER), defined as the substrate C : N ratio, at which decomposition switches from being C limited to being N limited, is thought to be a function of decomposer's stoichiometry and their carbon and sometimes nitrogen use efficiencies (Manzoni & Porporato 2009; Sinsabaugh *et al*. 2013):


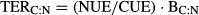
(1)

where NUE is microbial nitrogen use efficiency and B_C:N_ is microbial biomass C : N ratio. At common model assumptions of NUE = 1 and CUE = 0.5, TER_C:N_ would range from ∼16 to 30 (assuming microbial biomass C : N ratios range from 8 to 15). Litter with a higher C : N ratio than TER_C:N_ is thought to need external N to be decomposed. In a global data set of ∼2800 observations of decomposing litter, it has been shown, however, that TER_CN_ was positively correlated by power law to initial litter C : N ratio (Manzoni *et al*. 2008; Sinsabaugh *et al*. 2013), allowing even high C : N litter to be decomposed without external input of N (Moore *et al*. 2006; Parton *et al*. 2007). To explain this phenomenon, it has been concluded that [based on equation (1)] decomposers need to strongly decrease CUE with increasing N limitation, which is also in line with the concept of C overflow respiration at the microbial cell level (Manzoni & Porporato 2009). This implies, however, that a drastic waste of carbon would occur, wherever nutrients are limiting (Manzoni *et al*. 2008). Such a reduction in CUE with increasing N limitation is the prevailing assumption in almost all models and conceptual papers up to date (Schimel & Weintraub 2003; Manzoni *et al*. 2008; Manzoni & Porporato 2009; Sinsabaugh *et al*. 2013).

Our results here provide an alternative and contrasting explanation of this phenomenon. Decomposers in our model overcome the stoichiometric imbalance between the resource and their biomass through adjustments of the relative turnover rates of C- and N-rich pools, driven by a microbial-community response to resource stoichiometry. In fact, this community-driven mechanism fixes community CUE at a relatively high level, even when N concentration in the litter is low, instead of leading to its drastic decrease.

We thus propose that stoichiometric models need to account not only for substrate C : N ratios but also for the C : N ratio of bioavailable DOM, which is a result of relative differences in turnover rates of C- and N-rich pools *and* for the community-driven flexibility to adjust these relative differences. If we assume, for example, that the average N unit is reused *r* times more often per unit of time than the average C unit, the critical litter C : N ratio could subsequently be calculated as:


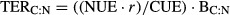
(2)

For incorporating a community-driven feedback loop between initial litter C : N ratio and N recycling, as our model suggest, *r* would need to scale with initial litter C : N ratio. This would effectively link TER_C:N_ to litter C : N ratio, as has been shown in the global litter decomposition data set (Manzoni *et al*. 2008), but without the need to severely reduce CUE, and thus increase C waste, with increasing nutrient limitation.

Bioavailable DOM, which we show to be critical in regulating the microbial response, may only be a fraction of the extractable DOM pool, rapidly consumed and thus difficult to measure in real systems. Interestingly, C : N ratios of bulk litter and extractable DOM turned out to be clearly decoupled during a 65-week-long litter decomposition experiment, supporting in general our model results (Fig. S1). Further research will be needed to identify suitable measurements of bioavailable DOM to evaluate this mechanism in nature. Notwithstanding this, our results clearly demonstrate that stoichiometric limitation of microbial decomposition may be different from what traditional mass balance equations predict. Although we implemented a mass balance-based overflow regulation at the cellular level in our model, the system behaviour at the community-scale turned out to be fundamentally different from the individual-scale (=physiological) regulation. The identification of such community-driven mechanisms is important for the accurate representation of stoichiometric relationships in biogeochemical models, which likely has a profound influence on the prediction of terrestrial C sequestration rates.
