# Supplementary material for: Microbial community dynamics alleviate stoichiometric constraints during litter decay
Source: Ecol Lett. 2014 Mar 17;17(6):680–90. doi: 10.1111/ele.12269 (PMC4315898; doi:10.1111/ele.12269)
Supplement: Supplementary file 1 [file ele0017-0680-sd1.pdf]

Microbial community dynamics alleviate stoichiometric constraints during litter decay

Supporting information

This file contains the following supporting information:

Figure S1: Decoupling of C:N ratios of bulk litter and dissolved material extracted from that litter measured during a litter decomposition experiment

Table S1: Overview of model parameters and their values as derived from the Bayesian Markov Chain Monte Carlo (MCMC) calibration

Appendix S1: Link between microbial cell size and turnover rates

Appendix S2: Enzyme production and kinetics

Appendix S3: Modeling diffusion at the micro-scale

Appendix S4: Calibration of the model by using a Bayesian approach (Markov-Chain-Monte-Carlo simulation)

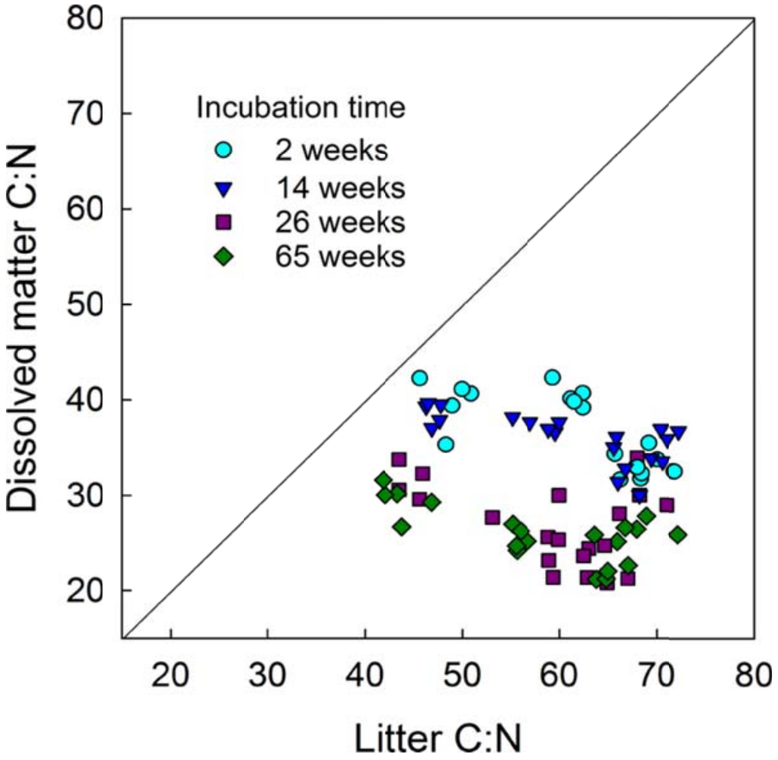

Figure S1. C:N ratios of litter versus C:N ratios of dissolved material extracted from that litter during litter decomposition. Data presented here are from an experimental study in which beech leaf litter from four different sampling sites in Austria (i.e. with varying initial C:N ratios) had been incubated under laboratory conditions for up to 65 weeks. Presented is total C:N ratio of litter (analyzed with an elemental analyzer, CN2000, Leco, St.Joseph, Michigan, USA) and the ratio of dissolved organic carbon to total dissolved nitrogen (extracted in 0.5 Mol K_2SO_4, analyzed by an TOC/TN analyzer, Shimadzu TOC-VCPH with TNM-1 module, Shimadzu, Vienna, Austria). Original data has partly been published in Mooshammer et al, 2012, and Leitner et al, 2012. Our analysis here shows that C:N ratio of dissolved material is both, constantly lower than C:N ratio of litter *and* decoupled from C:N ratio of litter.

Table S1: Overview of model parameters and their values as derived from the Bayesian Markov Chain Monte Carlo (MCMC) calibration (see Appendix S4).

Parameters	Description	Prior probability distribution	Result MCMC calibration (mean= parameter values used for scenario runs)	Unit
		range	*mean (c.v.)*	
**Enzyme kinetics***				
k_{cat}: number of enzymatic reactions catalyzed per enzyme (mol substrate-C decomposed per mol enzyme-C)				
k_{cat} PM	k_{cat} Plant material	0.1 – 1.2	0.66 (0.23)	timestep^{-1}
k_{cat} MR-C	k_{cat} C-rich microbial remains	0.1-10	1.89 (0.46)	timestep^{-1}
k_{cat} MR-N	k_{cat} N-rich microbial remains	0.1-1.2	0.9 (0.30)	timestep^{-1}
k_m: Half-saturation constant for substrate in one microsite				
k_m PM	k_m Plant material	0.01-1.2	0.29 (0.75)	fmol C
k_m MR-C	k_m C-rich microbial remains	0.01-1	0.28 (0.59)	fmol C
k_m MR-N	k_m N-rich microbial remains	0.01-1	0.25 (0.55)	fmol C
k_{enz}	First order rate constant for inactivation of enzymes	0.0001-0.05	0.036 (0.23)	timestep^{-1}
**Microbial physiology**				
maintResp	Maintainance respiration (fraction of biomass)	0.0001-0.06	0.008 (0.25)	timestep^{-1}
GE_Resp	Respiration for growth and enzyme production (fraction of carbon used for growth/enzyme prod.)	0.01-0.7	0.26 (0.45)	timestep^{-1}
U_{max}	Basic maximum uptake rate (as fraction of biomass, to be multiplied with individual surface:volume ratio, see Appendix S1)	0.003-0.03	0.0057 (0.17)	timestep^{-1}
m_f¶	Relation between mortality rate and the inverse of maximum biomass per MS	0.001-0.5	0.34 (0.25)	
**General**				
frLeach	Fraction of diffusing DOM that is lost by leaching	0.0001-0.012	0.0088 (0.21)	timestep^{-1}
**Functional groups**				

Group 1 (red)	*Assumptions: small bacterial cell size; no enzyme production; CN ratio = 6.21*†*;*			
Max cell size‡	Cell size at which cell division takes place	1-12	4.35 (0.30)	fmol C
enz fract	Fraction of DOM uptake (after deduction of maintainance respiration) that is invested in enzyme production	0	0	
Group 2 (blue)	*Assumptions: fungal cell size; plant degraders; CN ratio =12.22*†*;* *Ratio of enzyme produced for degradation of PM:MR-C:MR-N = 0.9:0:0.1.*			
Max cell size‡		80-150	97.23 (0.14)	fmol C
enz fract		0.02-0.2	0.15(0.17)	
Group 3 (green)	*Assumption: large bacteria cell size; secondary substrate degraders; CN ratio = 9.03*†*;* *Ratio of enzyme produced for degradation of PM:MR-C:MR-N = 0:0.5:0.5.*			
Max cell size‡		5-20	12.09 (0.20)	fmol C
enz fract		0.02-0.2	0.09 (0.41)	

Initial pool sizes in each microsite

enz amount	Initial amount of extracellular enzymes	0.5	fmol C
MRC	Initial amount of C-rich microbial remains	100	fmol C
MRN	Initial amount of N-rich microbial remains	30	fmol C
DOM	Initial amount of DOM	7	fmol C
DOM C:N	Initial DOM C:N ratio	8	
PS§	Initial amount of primary substrate C	8333	fmol C

"Prior" gives the parameter range defined *a priori* to the calibration (as a uniform probability distribution). "Result MCMC" presents mean and coefficient of variation (c.v.) of the 20% of entries with the highest likelihood in the MCMC chain (for details see Appendix S4). The parameter means resulting from the MCMC calibration were used as parameter settings for all model runs in this study. Settings for functional groups were only used for analyses presented in Fig.1, Fig.3 and Fig. 4, but not for Fig. 2 (for Fig. 2 settings see Table S2). One microsite = 1000μm^3. PM=Plant material. C-rich Microbial remains (MR-C) contains cell walls, lipids, starch (C:N ratio = 150). N-rich microbial remains (MR-N) contains proteins, DNA, RNA and inactive extracellular enzymes (C:N ratio = 5). One model time-step=3 hours.* Prior ranges for enzyme kinetics parameters (kcat and km) derived from (German *et al.* 2012). For achieving turnover number (kcat) of enzymes, we divided published vmax values by an estimated enzyme concentration of 1/10 of the microbial biomass in decomposing litter. †For ratios of cell compounds that produce the given C:N ratios see Table S2.‡ Max. number of cells per microsite: 30/max cell size for group 1 and 3,

respectively; 1 for group 2. Mortality rate=0.34/max. cell biomass per MS §Assuming leaf density: 500μg/mm^3 (Witkowski & Lamont 1991); water content: 60%, C content: 50% dry mass. ¶ Mortality rate is inverse to maximum biomass per microsite (=max cell size x max nr of cells per MS), assuming that larger cells invest more in defensive structures.

Appendix S1: Link between microbial cell size and turnover rates

In our model each functional group is characterized, amongst other traits, by its "maximum cell size" (MCS), which is the cell size at which a microbe divides and colonizes neighboring cells. All individuals of a functional group will thus have cell sizes distributed between half of MCS and MCS.

Microbial cell size is linked to maximum turnover rate of a functional group in our model. This is based on two assumptions:

(1) Effect of cell size on uptake rates

The smaller a microbe, the larger is its surface in relation to its volume and the more substrate it can thus take up in relation to its biomass. We thus assume that smaller cells can grow relatively faster compared to larger cells, and implemented this by making maximum uptake rates of bioavailable dissolved organic matter (DOM) a function of cell surface area:

Cell volume is derived from biomass-C,

$$V_{cell} = C_{BM}\, m_C\, 10$$

Assuming the density of a microbial cell to be 1000 fg fresh weight μm^{-3} (1g cm^{-3}); with C accounting for 10% of fresh weight (assuming 80% water content and C accounts for 50% of dry weight). (Romanova & Sazhin 2010). C_{BM} is biomass-C of the microbial cell in the model (in mol), m_C is the molar weight of C (12 g mol^{-1}), and V_{cell} is the volume of a microbial cell in cm^3.

Radius is derived from volume, assuming a spheric shape:

$$r = \sqrt[3]{\frac{3V}{4\pi}}$$

which is used to calculate the surface:volume ratio:

$$R_{sv} = 3/r$$

The basic maximum uptake Rate is multiplied with surface to volume ratio and biomass.

$$U_{pot} = U_{max}\, R_{sv}\, C_{BM}$$

Where U_{pot} is the potential amount of C that can be taken up by this microbe in one time step (given sufficient substrate availability). U_{max} is the basic maximum uptake rate, as defined in the general parameter settings (Table S1)

(2) Effect of cell size on mortality rates

We assume that species with larger cells invest more in structural and/or defensive cell compounds, which makes them more resistant against catastrophic death. Smaller cells have thus a higher chance to die than larger cells. We implemented this assumption by relating (stochastic) mortality rate inversely to MCS (Table S1).

$$m = \frac{1}{M_{CS}} m_f$$

Where m_f is the factor relating mortality rate to the inverse of maximum biomass (Table S1), M_{CS} is the maximum cell size (in fmol C) and m is the probability of a microbe to die in one time step.

Together, our assumptions imply that microbes with large cell sizes have a slower turnover rate compared to microbes with small cell sizes - as long as substrate is not limiting.

Appendix S2: Enzyme production and kinetics

Enzymatic breakdown

Extracellular enzymes break down complex substrate each time-step in each microsite according to Michaelis-Menten kinetics (Allison *et al.* 2010; Wang & Post 2013):

$$d_c = k_{cat}\, E\, \frac{S}{k_m + S}$$

d_c	Enzyme-catalysed break-down of complex substrate in one microsite (mol C transferred from the complex substrate to the bioavailable DOM pool per time step).
k_{cat}	Catalytic constant: number of enzymatic reactions catalyzed per time step per enzyme (mol substrate-C decomposed per mol enzyme-C).
S	Amount of complex substrate in this microsite (mol C)
k_m	Half saturation constant for substrate (mol C)
E	Amount of enzymes present in this microsite (mol C)

Implicit trade-off between enzyme production-growth

C and N for Enzyme production is deducted from C and N taken up each time step, and thus reduces resources available for growth.

Trade-off between enzyme production and cell size

With regard to a minimum of biomass C and N necessary to produce enzymes, we may assume that larger cells can afford enzyme production more easy compared to smaller cells. At least we could assume that very small cells are not able to produce enzymes as this needs a minimum of cytoplasma space.

Appendix S3: Modeling diffusion at the micro-scale

Diffusion is usually modeled by describing the average movement of particles on the macroscopic level for example based on Fick's Law. However, as our model operates on the microscopic level and is structured like a cellular automata, the most feasible way was to model diffusion on an atomic scale, i.e. simulating Brownian motion of individual particles. In a cellular automata approach of diffusion each molecule is given a probability to leave its grid cell per time step at random direction with a certain jump size (Kier *et al.* 1997). These parameters of thermic movement relate to the physical properties of the molecule, and the emergent outcome of this process, i.e. the phenomenon of diffusion on the macroscopic level, is usually (and indirectly) described by Fick's law.

At the atomic level, the average distance of each particle to its origin after a certain time (which is proportional to the diffusion coefficient) can be calculated from its "random walk":

$$R^2 = N\,j^2$$

where R^2 is the average square distance of each particle after a number N of random jumps with jumpsize j. For our purpose, N can be translated into $x\,t$, where t is the number of time steps and x is the probability that the particle will leave its grid cell in any one time step.

The diffusion coefficient, usually given in cm^2 per second, is defined as the square of the distance a particle will travel in one movement ($D = x\,j^2$). According to the rules of random walk explained above we can extrapolate the diffusion coefficient to our time step size by multiplying it with the number of hypothetical jumps (seconds) in one of our time steps (f.e. 30 min), which will give the mean square distance each particle has traveled in one time step:

$$D_m = D\,N$$

where N is the number of jumps within one time step in the model). From this adjusted diffusion coefficient we can infer the jumpsize for individual particles in each (discrete) model time step by:

$$j_m = \sqrt{D_m/x}$$

where j_m is the jumpsize per model time step, D_m is the diffusion coefficient per model time step, x is the probability of particle to leave its grid cell each time step.

For example, if we define that a particle will leave its microsite with probability 0.88 (which corresponds to 8/9: assuming that 1/9 of DOM stays in its microsite and the remaining 8/9 will distribute evenly among the 8 neighboring microsites), and a model time-step consists of 1 hour, a diffusion coefficient of $1.94*10^{-10}$ cm^2/sec (Tinker & Nye 2000) would translate to a jump size of 9.5 µm (~1 grid cell) in the model ($j_m = \sqrt{(D\ 3600)}/0.88$). Due to the discrete model space, the jump size has to be a multiple of one grid cell size. With these parameter values (j_m=10, x =0.88) individual particles will carry out random walk on the grid and will show a statistical average distance of 86 mm (8.6 grid cells) from their origin cell after 100 time steps ($L = \sqrt{(10^2\ 0.88\ 100)}$).

As a result, gradient dependent movement and mixing will emerge automatically on the grid scale, solely based on statistical movements of individual particles (Weimar & Boon 1994; Kier *et al.* 1997; Weimar 1997).

Appendix S4: Calibration of the model using a Bayesian approach (Markov-Chain-Monte-Carlo simulation)

Complex, process-based models are difficult to parameterize, because of a high degree of uncertainty associated with many of the parameters. We first aimed at defining reasonable ranges for the parameters from the literature (see footnotes for tables S1 and S2). After that we objected our parameter settings to a Bayesian calibration (using a Markov-chain Monte Carlo simulation), which has been proven a useful method for the parameterization of complex process-based models (Van Oijen *et al.* 2005).

In this approach, a given set of parameter values is evaluated by using a likelihood function which scores how well the model is able to reproduce empirical data. We used Markov-chain Monte Carlo simulation (MCMC) also known as Metropolis-Hastings random walk to walk through the multivariate parameter space (Van Oijen *et al.* 2005). We defined the boundaries of each of our 18 parameters (the “a priori” probability distribution of parameter values) based on the literature (see Table S1 and S2) or –where it was difficult to find reasonable measurements in the literature- based on assumptions. If there was little

previous knowledge about a parameter we defined a wide range of possible values for it in order not to constrain the calibration mechanism to unreasonable parameter settings. We also assumed “a priori” the presence of three functional microbial groups, one plant-degrader (fungal cell size, C:N ratio = 12.22), one microbial necromass degrader (large bacterial cell size, C:N ratio = 9.03) and one opportunistic bacterial group (small bacterial cell size, C:N ratio = 6.21) (Table S1 and S2).

The MCMC starts at a random point in the multi-dimensional parameter space, and then randomly moves from point to point based on a defined, maximum step-size. It calculates a likelihood score for the model output at each point. If the score is higher or slightly lower than the last accepted point, the point is accepted and recorded, and serves as a basis for calculating the next jump. Otherwise it is rejected and the last accepted point is recorded again (giving it more weight). In this way, a “chain” of parameter settings arises during the MCMC simulation, in which the likelihood initially increases, but then settles after a while to oscillate around a maximum value (Van Oijen *et al.* 2005).

The data set we used for calibration was derived from a laboratory litter decomposition study (Wanek *et al.* 2010; Keiblinger *et al.* 2012; Leitner *et al.* 2012; Mooshammer *et al.* 2012). Beech litter of four sampling sites in Austria differing in initial litter stoichiometry (mean±SE of litter C:N mass ratios: 41.8±0.8, 52.6±0.5, 57.9±0.6 and 60.0±0.7, respectively) had been incubated for up to 65 weeks. Measurements (litter C and N content, dissolved organic carbon and dissolved nitrogen, DIN, N gross mineralization, microbial respiration) had been carried out after 2,14,26 and 65 weeks. In this experiment, all litter types had been sterilized and re-inoculated with the same microbial community prior to the incubation. This resembles our model set-up, which does not account for different starting microbial communities, but rather aims at elucidating the influence of litter stoichiometry on community composition and decomposition. For the calibration we used data from two of the four sampling sites (initial litter C:N ratios 42 and 60). For each point in the parameter space the model was run in four replicates for both, initial litter C:N 42 and 60, and the mean was compared to the measured values. Doing so, we generated a MCMC chain of 20.000 entries (taking around five days computing time on a PC) with an acceptance rate of 1.4%.

We calculated mean and coefficient of variation for each parameter from the 20% of total chain entries (i.e. 4000) with the highest likelihood (Table S1). Mean values were used as parameter settings for all model analyses conducted for this study, except for the functional group settings in Fig. 2, which were varied for the analysis, and the one-group model displayed in Figure 4.

We finally let the model run at all of the four different initial litter C:N ratios and compared its output to the empirical data set. The model showed a good fit in the overall C and N dynamics (total C remaining and litter C:N ratios, R2=0.95 and 0.91, respectively, $p<0.0001$, Fig. 2). More specific pools and fluxes (such as gross N mineralization, microbial respiration, DIN) calculated by the model showed larger variations with respect to the empirical data, but were all in the same order of magnitude.

References

1.

Allison, S.D., Wallenstein, M.D. & Bradford, M. a. (2010). Soil-carbon response to warming dependent on microbial physiology. *Nat. Geosci.*, 3, 336–340.

2.

German, D.P., Marcelo, K.R.B., Stone, M.M. & Allison, S.D. (2012). The Michaelis-Menten kinetics of soil extracellular enzymes in response to temperature: a cross-latitudinal study. *Glob. Chang. Biol.*, 18, 1468–1479.

3.

Keiblinger, K.M., Schneider, T., Roschitzki, B., Schmid, E., Eberl, L., Hämmerle, I., *et al.* (2012). Effects of stoichiometry and temperature perturbations on beech leaf litter decomposition, enzyme activities and protein expression. *Biogeosciences*, 9, 4537–4551.

4.

Kier, L.B., Cheng, C.K., Testa, B. & Carrupt, P. a. (1997). A cellular automata model of diffusion in aqueous systems. *J. Pharm. Sci.*, 86, 774–8.

5.

Leitner, S., Wanek, W., Wild, B., Haemmerle, I., Kohl, L., Keiblinger, K.M., *et al.* (2012). Influence of litter chemistry and stoichiometry on glucan depolymerization during decomposition of beech (Fagus sylvatica L.) litter. *Soil Biol. Biochem.*, 50, 174–187.

6.

Mooshammer, M., Wanek, W., Schnecker, J., Wild, B., Leitner, S., Hofhansl, F., *et al.* (2012). Stoichiometric controls of nitrogen and phosphorus cycling in decomposing beech leaf litter. *Ecology*, 93, 770–82.

7.

Van Oijen, M., Rougier, J. & Smith, R. (2005). Bayesian calibration of process-based forest models: bridging the gap between models and data. *Tree Physiol.*, 25, 915–27.

8.

Romanova, N.D. & Sazhin, a. F. (2010). Relationships between the cell volume and the carbon content of bacteria. *Oceanology*, 50, 522–530.

9.

Tinker, P.. & Nye, P.. (2000). *Solute Movement in The Rhizosphere*. Oxford University Press, New York.

10.

Wanek, W., Mooshammer, M., Blöchl, A., Hanreich, A. & Richter, A. (2010). Determination of gross rates of amino acid production and immobilization in decomposing leaf litter by a novel 15N isotope pool dilution technique. *Soil Biol. Biochem.*, 42, 1293–1302.

11.

Wang, G. & Post, W.M. (2013). A note on the reverse Michaelis–Menten kinetics. *Soil Biol. Biochem.*, 57, 946–949.

12.

Weimar, J.R. (1997). Cellular automata for reaction-diffusion systems. *Parallel Comput.*, 23, 1699.

13.

Weimar, J.R. & Boon, J.-P. (1994). Class of cellular automata for reaction-diffusion systems. *Phys. Rev. E*, 49, 1749–1752.

14.

Witkowski, E.T.F. & Lamont, B.B. (1991). Leaf specific mass confounds leaf density and thickness. *Oecologia*, 486–493.
